# The observation that older men suffer from hip fracture at DXA *T*-scores higher than older women and a proposal of a new low BMD category, osteofrailia, for predicting fracture risk in older men

**DOI:** 10.1007/s00256-024-04793-2

**Published:** 2024-09-16

**Authors:** Yì Xiáng J. Wáng, Ben-Heng Xiao, Jason C. S. Leung, James F. Griffith, Maria Pilar Aparisi Gómez, Alberto Bazzocchi, Davide Diacinti, Wing P. Chan, Ali Guermazi, Timothy C. Y. Kwok

**Affiliations:** 1 Department of Imaging and Interventional Radiology, Faculty of Medicine, The Chinese University of Hong Kong, Shatin, New Territories, Hong Kong SAR China; 2 Jockey Club Centre for Osteoporosis Care and Control, Faculty of Medicine, The Chinese University of Hong Kong, Shatin, Hong Kong SAR China; 3 Department of Radiology, Auckland City Hospital, Auckland District Health Board, Auckland, New Zealand; 4 Department of Anatomy and Medical Imaging, Faculty of Medical and Health Sciences, The University of Auckland, Auckland, New Zealand; 5 Department of Radiology, IMSKE, Valencia, Spain; 6 Diagnostic and Interventional Radiology, IRCCS Istituto Ortopedico Rizzoli, Bologna, Italy; 7 Department of Diagnostic and Molecular Imaging, Radiology and Radiotherapy, University Foundation Hospital Tor Vergata, Rome, Italy; 8 Department of Radiology, Wan Fang Hospital, Taipei Medical University, Taipei, Taiwan; 9 Department of Radiology, School of Medicine, College of Medicine, Taipei Medical University, Taipei, Taiwan; 10 Department of Radiology, Boston University School of Medicine, Boston, MA USA; 11 Department of Medicine and Therapeutics, Faculty of Medicine, The Chinese University of Hong Kong, Shatin, New Territories, Hong Kong SAR China

**Keywords:** Osteoporosis, Bone mineral density (BMD), Reference database, *T*-score, Males

## Abstract

The clinical significance of osteoporosis lies in the occurrence of fragility fractures (FFx), and the most relevant fracture site is the hip. The *T*-score is defined as follows: (BMD_patient_–BMD_young adult mean_)/SDy_oung adult population_, where BMD is bone mineral density and SD is the standard deviation. When the femoral neck (FN) is measured in adult Caucasian women, a cutpoint value of patient BMD of 2.5 SD below the young adult mean BMD results in a prevalence the same as the lifetime risk of hip FFx for Caucasian women. The FN *T*-score criterion for classifying osteoporosis in older Caucasian men has been provisionally recommended to be − 2.5, but debates remain. Based on a systematic literature review, we noted that older men suffer from hip FFx at a FN *T*-score approximately 0.5–0.6 higher than older women. While the mean hip FFx FN *T*-score of around − 2.9 for women lies below − 2.5, the mean hip FF FN *T*-score of around − 2.33 for men lies above − 2.5. This is likely associated with that older male populations have a higher mean *T*-score than older female populations. We propose a new category of low BMD status, osteofrailia, for older Caucasian men with *T*-score ≤  − 2 (*T*-score ≤  − 2.1 for older Chinese men) who are likely to suffer from hip FFx. The group with *T*-score ≤  − 2 for older Caucasian men is comparable in prevalence to the group with *T*-score ≤  − 2.5 for older Caucasian women. However, older men in such category on average have only half the FFx risk as that of older women with osteoporotic *T*-score.

## Introduction

Osteoporosis is a systemic skeletal disease characterised by a reduction in bone mass and qualitative skeletal changes that cause an increase in bone fragility and a higher fracture risk. The clinical significance of osteoporosis lies in the occurrence of fragility fractures (FFx), and the most relevant fracture site is the hip. Among all FFx, hip fracture incurs the greatest morbidity, mortality, and costs. According to the criteria set by the 1994 World Health Organization (WHO) Study Group, the *T*-score is defined as follows: (BMD_patient_–BMD_young adult mean_)/SD_young adult population_, where BMD is bone mineral density and SD is the standard deviation [1]. For a variety of reasons, including differences in X-ray energy generation, bone edge detection algorithms, region of interest placement, and methods of calibration, BMD by DXA (dual-energy X-ray absorptiometry) in g/cm^2^ differs among DXA manufacturers. To avoid the confusion that would result from instrument-specific numerical BMD cutpoint values, the *T*-score concept was proposed, whereby each patient’s value is compared with a young normative database generated on the same device. Although BMD is recognized as a continuous risk factor for fracture (i.e., no natural fracture threshold exists), operational ranges for the *T*-score were initially proposed for epidemiologic purposes. When the femoral neck (FN) is measured in adult Caucasian women, a cutpoint value of patient BMD of 2.5 SD below the young adult mean BMD results in a prevalence of osteoporosis for those aged ≥ 50 years of about 16.2%, the same as the lifetime risk of hip FFx for Caucasian women [1]. This definition is the cornerstone of densitometric osteoporosis classification. For US Caucasians, the BMD reference range provided by the Third National Health and Nutrition Examination Survey (NHANES III) database is commonly used for calculating the *T*-scores [2]. Because hip FFx typically necessitates hospitalization, data on their incidence is more reliable than data on other types of fractures. Following the 1994 WHO definition, densitometric osteoporosis prevalence among a specific population should be in proportion to its relative osteoporotic fracture risk with Caucasian female data as reference [1, 3]. On the other hand, *Z*-score is the number of SD a patient’s BMD differs from an average person of the same age, gender, and ethnicity. Because BMD decreases with age, it is possible to have a *Z*-score close to zero but still have low BMD; thus, *Z*-score is more used for diagnosing secondary osteoporosis, especially for children and younger adults. The *Z*-score indicates whether additional reasons for bone loss (besides aging) are likely.

The WHO Study Group did not establish a guideline for the diagnosis of osteoporosis in men. Since the establishment of the FN *T*-score criterion for classifying osteoporosis in older women, there have been many debates on how to define the FN *T*-score criterion for classifying osteoporosis in older men [4–10]. Until now, the FN *T*-score criterion for classifying osteoporosis in older men has been provisionally recommended as − 2.5, the same as recommended for women [11, 12]. However, men have very different bone property features compared to those of women. Men have a higher FN BMD_young adult mean_ value and a wider FN SD_young adult population_, and older men suffer from FFx at a higher BMD than women [13, 14]. Considering these factors, intuitively it would be a rare coincidence if FN *T*-score of − 2.5 in older men has a clinical relevance being equivalent to FN *T*-score of − 2.5 in older women. Following the initial debates on the definition of osteoporosis *T*-score for men, more evidence has been recently published. We have recently noted that older men suffer from hip FFx at a higher *T*-score than older women [15, 16]. In this article, with a focus on the FN, we included more data and expanded the analyses, and we further propose that FN *T*-score of − 2.0 should be a better cutpoint value to predict older Caucasian men (− 2.1 for Chinese men) at an elevated risk of hip FFx.

### Cross-sectional studies show, when an acute hip FFx occurs, FN and/or TH T-score measures approximately 0.5–0.6 higher in male patients than in female patients

**Fig. 1:**
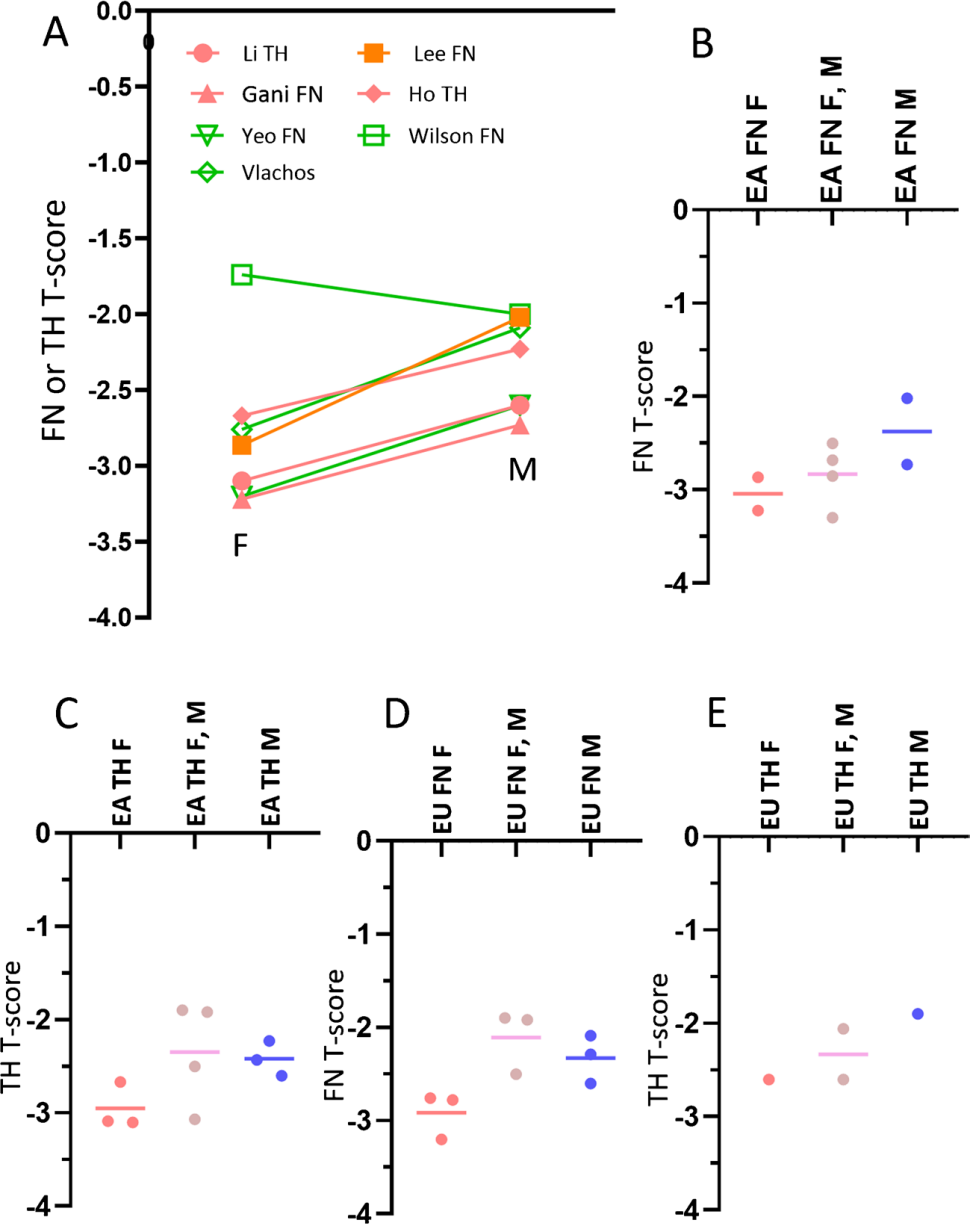
A comparison of femoral neck (FN) and total hip (TH) *T*-scores of female (F) and male (M) acute hip FFx patients. **A** Mean FN or TH *T*-score of M and F patients. Data are from Wilson et al. [17], Yeo et al. [18], Vlachos et al. [19], Lee et al. [20], Gani et al. [21], Ho et al. [22], and Li et al. [23]. Except the data of Wilson et al. [17], all other six groups of data show a higher FN or TH *T*-score in men than in women. For the study of Wilson et al. [17], while the FN *T*-score was higher in women, the study cases’ LS *T*-score and wrist *T*-score were higher in men than in women. Yeo et al. [18] and Gani et al. [21] also presented TH *T*-score, with a similar trend as shown in this graph. Caucasian data in green color and East Asian data in pink color. **B** FN *T*-score of East Asian (EA) patients. Each dot represents the mean *T*-score of one study respectively [20, 21, 24–27]. For the data from Lee et al. [20] and Gani et al. [21], M and F patients are presented separately. Mixed gender patient data are from Hey et al. [24], Li et al. [25], Kanno et al. [26], and Xu et al. [27]. **C** TH *T*-score of EA patients. Each dot represents the mean *T*-score of one study respectively [21–24, 27–29]. For the data from Gani et al. [21], Ho et al. [22], and Li et al. [23], M and F patients are presented separately. Mixed gender patient data are from Hey et al. [24], Xu et al. [27], Cha et al. [28], and Yamamoto et al. [29]. **D** FN *T*-score of European (EU) patients. Each dot represents the mean *T*-score of one study respectively [18, 19, 30–32]. For the data from Yeo et al. [18] and Vlachos et al. [19], M and F patients are presented separately. Mixed gender patient data are from Heetveld et al. [30], Valentini et al. [31], and Amar et al. [32]. **E** TH *T*-score of EU patients. Each dot represents the mean *T*-score of one study respectively [18, 32, 33]. For the data from Yeo et al. [18], M and F patients are presented separately. Mixed gender patient data are from Amar et al. [32] and Ganhão et al. [33]. In **B, C, D, E,** M patients: blue dots; mixed gender patients: gray dots; F patients: pink dots. Bars: mean values, with each study giving an equal weight (i.e., sample size for each study was not weighted). FFx, fragility fracture

We systematically searched literature reports on DXA *T*-score results measured at the timepoint when a hip FFx occurred. On April 15, 2024, a structured literature search on https://pubmed.ncbi.nlm.nih.gov/ was conducted using the keywords combination of “((hip OR femur OR femoral) AND fracture) AND *T*-score.” We aimed to only include studies concerning the contralateral FN/TH DXA BMD measured right after the time of fracture, and we only included reports with data for both female patients and male patients. Additional exclusion criteria were (1) articles concerned with patients’ group-wise under a specific anti-osteoporotic treatment regime, (2) articles concerned with specific types of patients such as those with diabetes mellitus type 2, (3) articles only concerned with hip re-fracture patients, (4) articles concerned with atypical femur fracture, (5) articles concerned with femoral head subchondral insufficiency fracture, and (6) study cohorts with fewer than ten cases. For articles from East Asia, we only included studies which used a local or an East Asian gender-specific BMD reference to calculate the *T*-score. We were able to identify seven studies that reported both women’s and men’s FN and/or TH (total hip) data separately [17–23] and ten studies that reported women’s and men’s FN and/or TH data together [24–33]. All Caucasian data are from Europe, and we assume that most of their older patients were Caucasians. East Asian studies were from South Korea, Singapore, Hong Kong, and China mainland. The results of these 17 articles are shown in Fig. 1.

Figure 1A shows, out of seven studies, six studies showed a lower FN or TH *T*-score in female hip FFx patients than among male patients (two studies reported both FN and TH values, with FN and TH values showing the same trend). In the seven studies shown in Fig. 1A, three studies, all showing a higher FN and/or TH *T*-score for males, reported the *p*-value for male vs. female *T*-score comparison. Yeo et al. [18] described a *p*-value of < 0.073 for TH *T*-score and a *p*-value of < 0.123 for the FN *T*-score. Vlachos et al. [19] described a *p*-value of < 0.020 for FN *T*-score. Lee et al. [20] described a *p*-value of < 0.001 for FN *T*-score. Figure 1 B–E separately lists the results for East Asians and for Europeans, and the results for FN and for TH, with the results consistently showing a lower FN/TH *T*-score among female hip FFx patients than among male patients. If men suffer from hip FFx at a higher *T*-score than women, then a mixed-gender cohort would have a higher FN or TH *T*-score than the female patients. Indeed, Fig. 1B–E shows, while individual studies reported varying results, the mean trends of the studies are consistent that the mixed-gender cohorts had a higher *T*-score than those of the females only data. The results in Fig. 1 are also supported by other reports. For example, Wong et al. [34] reported an Australian study with 47 female and 16 male patients suffered from various low-energy trauma fractures. FN BMD was measured within 12 months of the hip FFx incident, and the mean FN *T*-score was − 2.78 for female patients and − 2.29 for male patients.

## Male patients suffer from FFx at higher FN and/or TH BMD than female patients

**Fig. 2:**
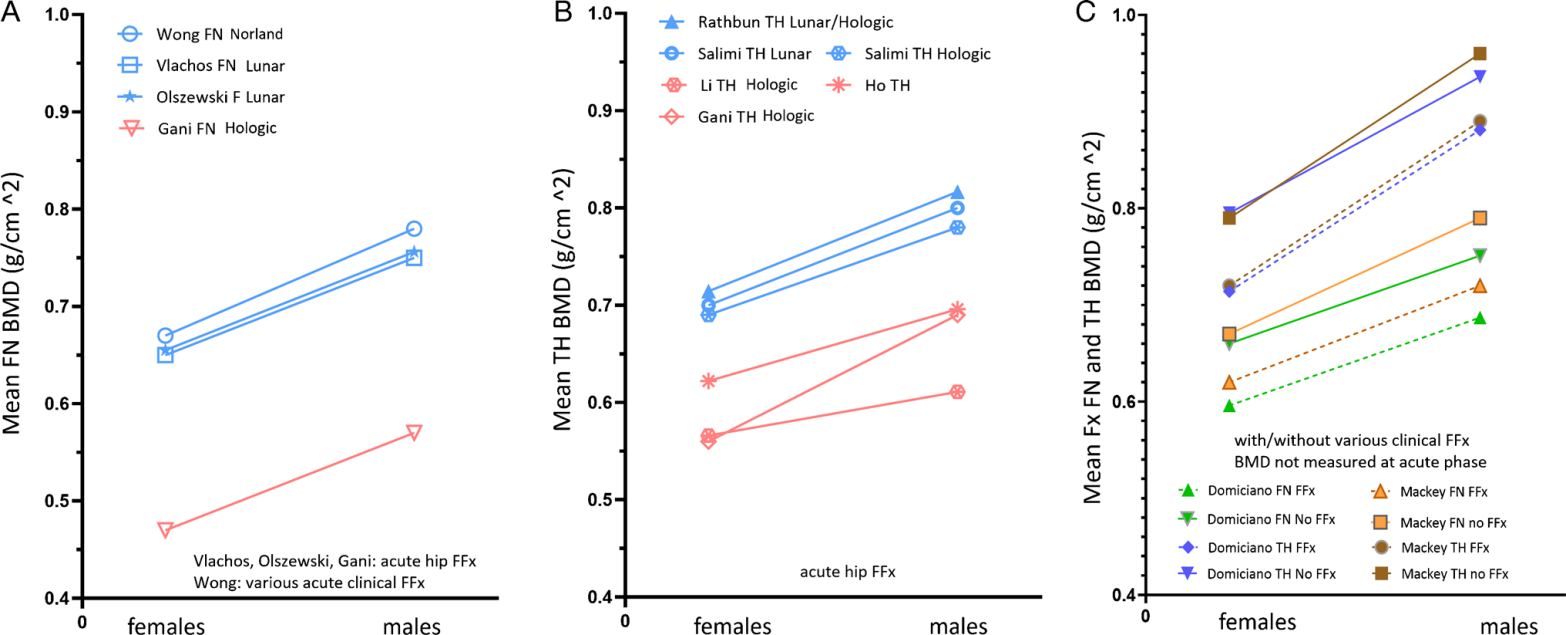
Older men sustain FFx at higher BMDs than older women. *Y*-axis is the mean BMD of patients when FFx occurred. **A** Femoral neck (FN) BMD for male and female patients with acute FFx. Data from Vlachos et al. [19], Gani et al. [21], Wong et al. [34], and Olszewski et al. [35]. **B** Total hip (TH) BMD for male and female patients with acute hip FFx. Data from Gani et al. [21], Ho et al. [22], Li et al. [23], Rathbun et al. [36] (> 90% the study cases being USA Caucasians), and Salimi et al. [37] (USA data, measures by both Lunar DXA scanner and Hologic DXA scanner provided). In** A** and **B** East Asian data in pink color, and other data (from Europe, Australia, and USA) are in blue. **C** A random selection of two reports with the data of Mackey et al. [38] with patients of various clinical FFx (Hologic DXA scanner) and Domiciano et al. [39] with patients of various non-vertebral clinical FFx (Hologic DXA scanner). FFx, fragility fracture

For the studies listed in Fig. 1, four studies also reported FN and/or TH BMD values. We added another random selection of six studies as described in reference [14], and the results are together shown in Fig. 2 [19, 21–23, 34–39]. A random selection of the Australian data of Wong et al. [34] is used to show that different manufacturers’ DXA scanners gave an almost identical trend. Figure 2 shows men suffer from FFx at a notably higher FN/TH BMD than women. In Fig. 2A, Caucasian male patients had approximately 0.1 g/cm^2^ higher FN BMD than women [19, 34, 35], which is equivalent to approximately higher 0.73 FN *T*-score (SD_young adult population_ = 0.137 g/cm^2^) for Caucasian men [2]. Figure 2C shows male–female differences in FFx FN/TH BMD tend to parallel the male–female BMD differences of the subjects without FFx.

For the East Asian results shown in Fig. 1, an East Asian male BMD reference database was used to calculate the *T*-score for male patients. The use of Asian-specific reference data was recommended by the ISCD Asia–Pacific Region 2010 Consensus [40]. For the Caucasian results shown in Fig. 1, it was not described in the original articles which BMD reference database was used to calculate the *T*-score. The International Society for Clinical Densitometry (ISCD) 2001 position statement recommended using a young normal Caucasian database to calculate *T*-scores, regardless of race, using a male database for men and a female database for women [10]. However, subsequently, the 2013 ISCD consensus recommended a Caucasian female reference database for *T*-score calculation in men and states: “*A uniform Caucasian (non-race adjusted) female reference database should be used to calculate T-scores for men of all ethnic groups*” [41]. This statement was recommended for the US populations, and no position was taken with respect to BMD reference data or ethnicity-matching outside of the USA. This recommendation has generated controversies, and it remains a common practice that a Caucasian male BMD reference database is used to calculate the *T*-score for Caucasian men [6]. To our knowledge, most of the European sites use a male BMD reference database to calculate the *T*-score for men. This point (i.e., male BMD reference databases were used) is also suggested in Fig. 1A and Fig. 2A, with the differences between female and male patient values were consistent across the six studies. This is also supported by the fact that, as shown in Fig. 2A, male patients had 0.1 g/cm^2^ higher FN BMD, which is approximately 0.73 higher FN *T*-score units. A patient’s *T*-score result depends on the BMD reference database used to provide the values of BMD_young normal mean_ and SD_young normal population_. Given a male patient’s BMD value, if the *T*-score were calculated by a female BMD reference database, then the Caucasian male *T*-scores would be higher (healthier), and the male–female difference in *T*-score would be even larger.

### Prospective Chinese studies results show that older men suffered a hip FFx during follow-up had a higher baseline FN T-score than that of older women suffered a hip FFx during follow-up

Osteoporotic fractures in men (MrOS) and women (MsOS) Hong Kong represent the first large-scale prospective cohort studies conducted on bone health in East Asians [42–44]. At baseline, 2000 Chinese men and 2000 Chinese women ≥ 65 years were recruited from the local communities from August 2001 to March 2003, to determine the relationship between anthropometric, lifestyle, medical, and other factors with bone mineral density (BMD) measured at the hip and spine. The subjects had a baseline mean age of 72.3 years (range, 65–92 years) for men and 72.5 years (range, 65–98 years) for women. The recruitment criteria were structured so that the study results would represent similarly aged community-dwelling ethnic Chinese men and women in Hong Kong. All subjects were able to walk without assistance, without bilateral hip replacement, and have the potential to survive the duration of primary study for at least 4 years as judged by their pre-existing medical status. Men and women of similar age and from the same community-based population were investigated using the same methodology, thereby enabling a comparison of the results for men and for women.

**Fig. 3:**
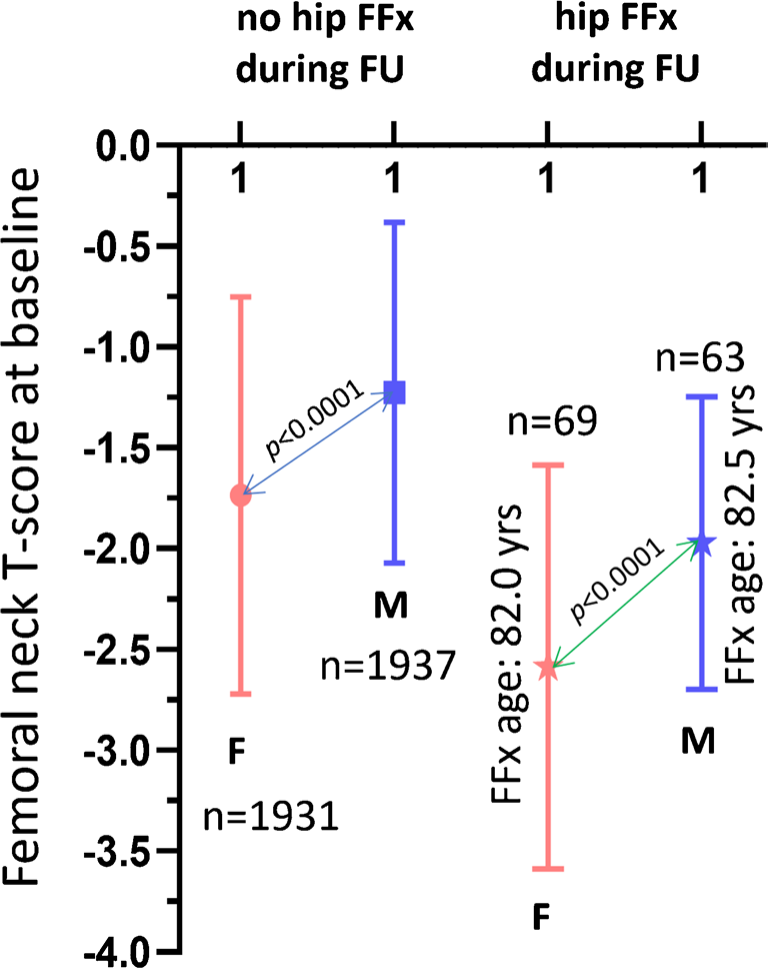
Males who suffered hip FFx during follow-up have a higher baseline femoral neck (FN) *T*-score than those of females who suffered hip FFx during follow-up. Data from MrOS and MsOS Hong Kong studies. It can be seen that the male–female difference in baseline FN *T*-score for subjects with hip FFx during follow-up parallels the male–female difference for the subjects without hip FFx during follow-up (lines with arrow at both ends). Males’ *T*-score was significantly higher than that of females both for the no FFx groups and the FFx groups (both, *p* < 0.0001). FFx, fragility fracture

Two thousand Chinese women (baseline age, 72.5 years) were followed up for 8.8 ± 1.5 years, and 69 FFx were recorded. Two thousand Chinese men (baseline age, 72.3 years) were followed up for 9.9 ± 2.8 years, and 63 hip FFx were recorded. With local female and male BMD references, the female and male FFx patients had a baseline *T*-score of − 2.59 ± 1.00 and − 1.97 ± 0.72 (*p* < 0.0001), respectively, and the mean age at hip FF was 82.0 and 82.5 years respectively (Fig. 3). These results are consistent with the results in Fig. 1, with male patients having a mean baseline FN *T*-score 0.62 higher than those of female patients.

## A proposal of a new low BMD category for predicting hip fracture risk in older men

**Fig. 4:**
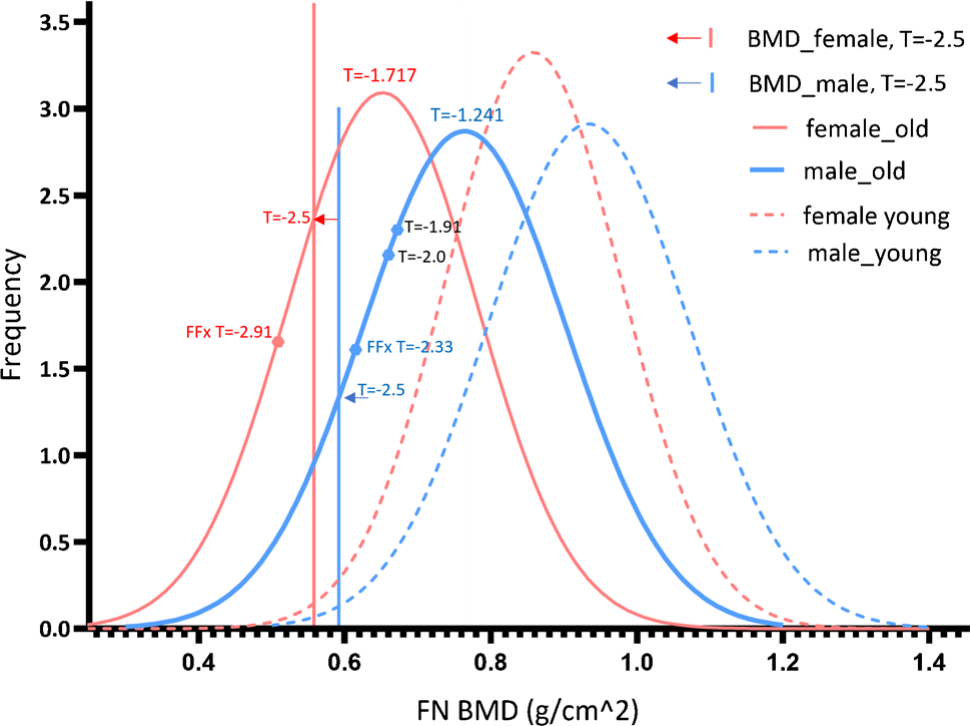
The femoral neck (FN) BMD distribution (Hologic DXA scanner) of US Caucasians and thresholds to define osteoporosis. The BMD distribution data of both young population and older population (≥ 50 years) are from Looker et al. [2]. The mean FN *T*-score among US older Caucasian community men was − 1.241, the mean FN *T*-score among US older Caucasian community women is − 1.717, and the *T*-score difference is 0.476. Red arrow: the *T*-score to define osteoporosis in women (prevalence = 23.4%). Blue arrow: the *T*-score to define osteoporosis in men (prevalence = 10.8%). *T*-score =  − 2.91 and *T*-score =  − 2.33 are the approximately estimated mean hip fracture *T*-scores for women and men respectively (estimated based on the mean of data of Yeo et al. [18] and Vlachos et al. [19], which is also consistent with the data of Wong et al. [34])

Evidence suggests that, for both East Asians and Caucasians, the prevalence of hip FFx in elderly women is approximately twice that of older men. For example, the review by Rapp et al. [45] concluded that the age-standardized difference in hip FFx prevalence between women and men has a ratio of about 2:1 in most countries of the world. Gong et al. [46] assessed hip fracture incidence in seven geographical regions of China, which involved 238,230 hip fracture patients aged 65 years or older treated between 2013 and 2016, and reported a ratio of 1.95. The population-based Dubbo study in Australia documented the incidence of all symptomatic fractures from 1989 to 1992 in a predominantly Caucasian population ≥ 60 years old. It was estimated that the residual lifetime fracture risk in a person aged 60 years with average life expectancy is 29% for men and 56% for women [47]. Fang et al. [48] analyzed the hip fracture hospitalization rates for patients aged 50 and older in New York City from 1988 to 2002. They reported annual age-adjusted hip fracture hospitalization rates per 100,000 among Caucasian women and men and 459 and 230 respectively. According to these data, the prevalence of osteoporosis of older men would be approximately half of that of older women. Figure 4 illustrates the BMD distribution of US Caucasians NHANES III data based on the report of Looker et al. [2]. A FN *T*-score cutpoint of − 2.5 in older men defines 10.8% of the older male population, and a FN *T*-score cutpoint of − 2.5 in older women defines 23.4% of the older female population. Faulkner and Orwoll [9] also described that a FN *T*-score cutpoint of − 2.5 in older US Caucasian men defines 9.0% of the older male population. Therefore, the FN *T*-score of cutpoint − 2.5 for both older women and men satisfies the concept that the prevalence of densitometrically defined osteoporosis in older men is approximately half of that in older women.

Figure 1D shows that Caucasian older men suffer from hip FFx at 0.59 FN *T*-score higher than women, which is also similar to East Asian data (Fig. 1B, Fig. 3). If other factors being equal, an older Caucasian man with a FN *T*-score of − 1.9 will be similar in hip FFx risk to an older Caucasian woman with a FN *T*-score of − 2.5, suggesting that a FN *T*-score of − 1.9 (or simply − 2.0 for convenience) should be a more suitable cutpoint value to identify FFx high-risk older Caucasian men. In Fig. 4, while the mean female hip FFx FN *T*-score of − 2.91 lies substantially below the female osteoporosis *T*-score threshold which is reasonable from a diagnostic screening point of view, that the mean male hip FF FN *T*-score of − 2.33 lies substantially above the male osteoporosis *T*-score threshold is less practical for diagnostic screening.

If we use a FN *T*-score ≤  − 2.0 to define “densitometric osteoporosis,” then the “densitometric osteoporosis” prevalence in older Caucasian men would be 22.7%. This is in conflict with the concept that the prevalence of FN densitometric osteoporosis among older men, which is defined as being equal to the prevalence of hip FFx among older men, is approximately half of that of older women. Men suffer from hip FFx also with a greater variation of *T*-score and BMD (Table 1). Relative to female data, male FFx *T*-score would be “*more scattered*” around − 2.33 (Table 1). We suggest that FFx characteristics between older men and older women are not only quantitatively different, but also qualitatively different. We propose a new category of low BMD status with *T*-score ≤  − 2 for older Caucasian men, “osteofrailia,” to define the group of older Caucasian men who are likely to suffer from hip FFx.

**Table 1:** Male female differences of standard deviation (SD) of NHANES III FN and TH *T*-score of various reports

Sources	Standard deviation of measures					
	M FN	F FN	M TH	F TH	M LS	F LS
NHANES III [2] ^*y*^	0.137^**‡**^	0.120				
NHANES III [2] ^*o*^	0.139^**‡**^	0.129				
Hong Kong Chinese [49] ^*y*^	0.130^**‡**^	0.100	0.140 ^**‡**^	0.110	0.110	0.100
Hong Kong Chinese [49] ^*o*^	0.115^**‡**^	0.107	0.136 ^**‡**^	0.115	0.162‡	0.140
Korean [50] ^*y*^	0.140^**‡**^	0.100			0.120	0.120
Korean [50] ^*o*^	0.130^**‡**^	0.125			0.174‡	0.159
Wilson et al. [17] ^#^	0.93	1.39			1.20	1.69
Vlachos et al. [19] ^#^	1.37^**‡**^	0.90				
Lee et al. [20] ^#^	1.12^**‡**^	0.92			1.71^**‡**^	1.48
Gani et al. [21] ^#^	0.90	0.89	0.97	1.01	1.55^**‡**^	1.33
Li et al. [23] ^#^			1.29^**‡**^	0.98	1.32^**‡**^	0.85

^y^N BMD SD of young adult community population

^o^FN BMD SD of older community population (older population of Hong Kong data had age: ≥ 60 years, NHANES III ≥ 50 years)

^#^*T*-score SD of patients with hip FFx

^‡^Male data have a larger SD than female data

For the data listed in Fig. 1A, Yeo et al. [18] and Ho et al. [22] did not report SD. The result of Wilson et al. is an outlier that the SDs of FN and LS of males were much smaller than FN SD of females

*FN* femoral neck, *TH* total hip, *LS* lumbar spine, *F* female, *M* male

**Fig. 5:**
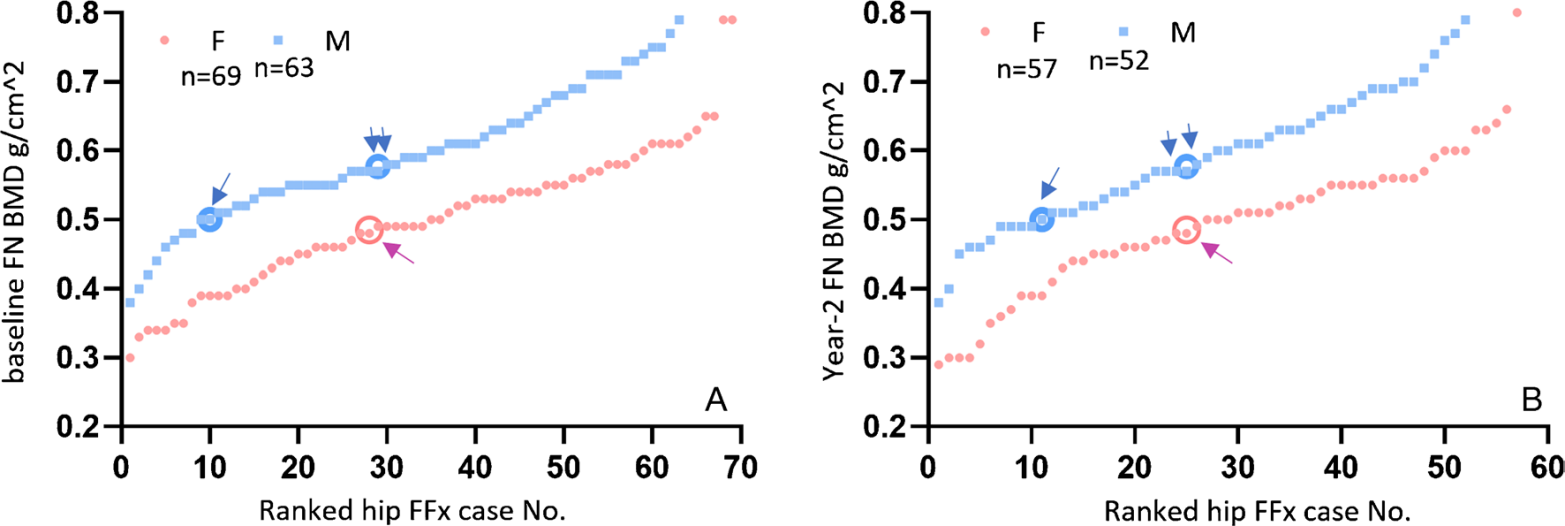
Different BMD thresholds to predict hip FFx during follow-up. Data from MrOS and MsOS Hong Kong studies. Sixty-nine hip FFX were recorded for women and 63 hip FFx were recorded for men. Each dot represents a case with hip FFx. *Y*-axis: femoral neck (FN) BMD measured at baseline (**A**) and at year 2 follow-up (**B**). *X*-axis: FFx cases ranked from the lowest BMD to highest BMD. Single blue arrow: BMD values corresponding to *T*-score threshold of − 2.7 for osteoporosis in Chinese men and women, double blue arrows: BMD value corresponding to *T*-score threshold of − 2.1. This graph shows baseline BMD measurement and year 2 BMD measurement show similar patterns with association of hip FFx (mitigating the possibility of major measurement imprecision errors). F, female; M, male; FFx, fragility fracture

With MrOS (Hong Kong) and MsOS (Hong Kong) data, Fig. 5 A, B shows the BMD values at baseline and at year 2 follow-up, respectively. Due to the lower prevalence of FFx among Chinese men and women than among Caucasians, it has been proposed that FN *T*-score ≤  − 2.7 is to classify osteoporosis [51]. Figure 5 shows that if we use FN *T*-score ≤  − 2.7 to determine male study subjects, then only a small portion of hip FFx cases during the 9.9 years’ follow-up (15.9%, 10/63) would be predicted. If we use FN *T*-score ≤  − 2.1, which will be equivalent to the Caucasian male *T*-score of 2.0 [52], to determine male study subjects, then 46.0% (29/63) of hip FFx cases would be predicted. For females, if we use *T*-score ≤  − 2.7 to determine study subjects, then 44.9% (31/69) of hip FF cases would be predicted during the 8.8 years’ follow-up. For men, osteoporosis threshold and osteofrailia threshold counted for 3.35% and 15.5% of the study baseline population. For women, osteoporosis threshold counted for 16.9% of the study baseline population. Therefore, “osteofrailia” for men may offer a comparable category in prevalence as osteoporosis for women (Table 2). Though the prevalence of hip FFx is substantially lower among men than among women, multiple reports described that, once hip FFx occurs, the post-fracture mortality is higher among men than among women. In a study based on 2000–2011 data of California, USA, males were nearly two times more likely to die within 30 days of a hip FFx than females (odds ratio 1.79, 95% CI 1.72, 1.86, *p* < 0.001) and were also more likely to die at other assessed time points (90 and 365 days) [53]. In a Danish published in 2010, compared with the general older population, the 1-year mortality after hip fracture increased by 27.2% in male patients and by 17.1% in female patients [54]. In a Hong Kong study published in 2016, mortality at 1 year after hip fracture was 27% in males and 15% in females [55]. In a study of the mainland China published in 2022, mortality at 1 year after hip fracture was 19.63% in males and 13.46% in females [56]. In addition, some studies suggest that males tend to suffer from hip FFx at a younger age than females [18–21, 35, 56].

**Table 2:** Outcome agreement between women FN *T*-score ≤  − 2.5 and men FN *T*-score ≤  − 2.0

Criteria	Women *T*-score ≤ − 2.5 ^#^	Men *T*-score ≤ − 2.0 ^#^
Disease prevalence, US Caucasian older population (≥ 50 years) [2]	23.4% ^‡^	22.7% ^‡^
Disease prevalence, Chinese older population (men, 72.3 years, women: 72.5 yrs) ^¶^	16.9% (338/2000)	15.5% (310/20000)
Baseline FN *T*-score to predict hip FFx during follow-up) ^¶¶^	44.9% (31/69) predicted	46.0% (29/63) predicted

^#^For Caucasians, cutpoint values of women *T*-score ≤  − 2.5 and men *T*-score ≤  − 2.0 are taken. For Chinese, according to Wang et al. [51], revised cutpoint values of women *T*-score ≤  − 2.7 and men *T*-score ≤  − 2.1 are taken

^‡^Estimated from the Third National Health and Nutrition Examination Survey (NHANES III) database[2]

^¶^MrOS and MsOS Hong Kong studies Chinese baseline data

^¶¶^MrOS and MsOS Hong Kong Chinese studies data, with a mean follow-up duration of 9.9 years for men and 8.8 years for women

Though the category of osteopenia (*T*-score ≤ 1) has been proposed, there is no biological or epidemiological rationale for the *T*-score threshold of − 1 [57]. The original intention of the WHO was to choose a threshold that would make osteopenia uncommon at the time of menopause. Actually, the prevalence of osteopenia is much higher [57]. According to the data of Wright et al. [58], osteopenia prevalence in US population over the age of 50 years is 51.4% for Caucasian women and 36.0% for Caucasian men. This makes osteopenia a less relevant diagnosis for patients [57].

## Implications for TH *T*-score and LS osteofrailia *T*-score classifications

**Fig. 6:**
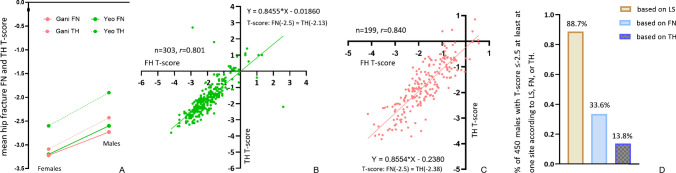
Correlation between femoral neck (FN) *T*-score and total hip (TH) *T*-score. **A** Mean hip FFx FN and TH *T*-scores data from Yeo et al. [18] (Caucasian men and women) and Gani et al*.* [21] (Singaporean men and women). **B** Italian Caucasian women data (age, 73.6 ± 6.1 years) from Wang et al. [59], with Pearson *r* of 0.801 (*p* < 0.0001). **C** Chinese women data (age, 74.1 ± 6.14 years) from Wang et al. [60], with Pearson* r* of 0.840 (*p* < 0.0001). **D** Data from Viapiana et al. [61] with a total of 450 osteoporotic Italian older men (age, 67.5 ± 9.6 years)

The FN *T*-score and TH *T*-score are known to be highly correlated. Data from Fig. 1 and Fig. 2 suggest that the FFx TH BMD/*T*-score difference between older men and older women is comparable to the FFx FN BMD/*T*-score difference. Figure 6A shows, for the studies of Yeo et al. [18] (Caucasian men and women) and Gani et al. [21] (Singaporean men and women), hip FFx TH *T*-score measured higher than hip FFx FN *T*-score. Figure 6B shows [59], with a data of 303 Italian Caucasian women, the correlation between FN *T*-score and TH *T*-score was 0.801, and FN *T*-score of − 2.5 corresponds to TH *T*-score of − 2.13. Figure 6C shows [60], with a data of 199 Chinese women, the correlation of FN *T*-score and TH *T*-score was 0.84, and FN *T*-score of − 2.5 corresponds to TH *T*-score of − 2.38. Figure 6D shows [61], for a total of 450 osteoporotic Italian older men, FN *T*-score defines 33.6% of the subjects being osteoporotic, while TH *T*-score defines 13.8% of the subjects being osteoporotic, thus suggesting that TH *T*-score was also higher than FN *T*-score. Therefore, we could use a threshold higher than − 2.0, such as − 1.7, to define the osteofrailia TH *T*-score. We suggest that, when TH is reported alone, a threshold of ≤  − 1.7 is used; when FN and TH are reported together and with the lower *T*-score to define osteofrailia, then a TH threshold of ≤  − 2.0 is used, knowing that the FN *T*-score will be a more sensitive biomarker in the majority of patients.

**Fig. 7:**
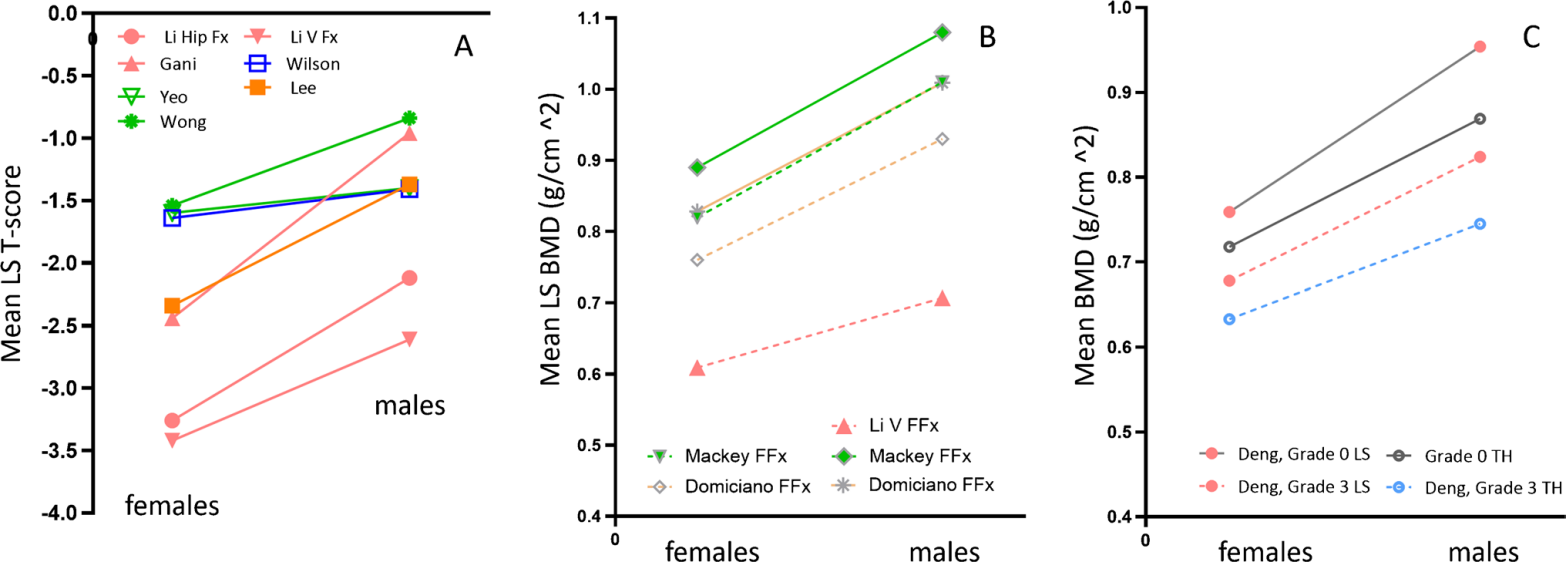
Male patients suffer from FFx at a higher lumbar spine (LS) *T*-score and BMD than female patients. **A** LS T-score data with the studies listed in Fig. 1A (hip FFx), the data of Li et al*.* [23] with clinical vertebral FFx (Li VFx), and the data of Wong et al. [34] with patients of various FFx. East Asian data in pink color. **B** LS BMD data of Li et al. [23] with clinical vertebral FFx, and a random selection of two reports with the data of Mackey et al. [38] reporting patients of various FFx and Domiciano et al. [39] reporting patients of non-vertebral FFx. **C** BMD data of Deng et al. [65] on radiographic vertebral FFx of community subjects who had or did not have radiographic grade 3 vertebral FFx. Grade 0: no FFx; grade 3: FFx with > 40% vertebral height loss. FFx, fragility fracture

Figure 7 suggests that older men suffer from FFx at a higher LS *T*-score than older women. However, it is more difficult to define the osteofrailia LS *T*-score. The correlation between LS *T*-score and FN *T*-score is known to be not as strong as that between TH *T*-score and FN *T*-score. In the MrOS (Hong Kong) baseline study, the correlation between LS *T*-score and FN *T*-score was moderate with Pearson *r* of 0.651 (*n* = 1961 male cases). The increasing prevalence of osteoarthritis with age likely lead to overestimate LS BMD. On the other hand, measurement of the LS BMD is more sensitive in some cases, especially in early postmenopausal women in whom more rapid decreases in BMD occur at the LS site (enriched in cancellous bone) than at the hip. Though primary osteoporosis is a systematic disease that affects the whole skeleton, it has been demonstrated that FN BMD reduction best predicts hip FFx, and LS BMD reduction best predicts vertebral FFx [62, 63]. Figure 1 and Fig. 7 together show that, when acute hip FFx occur, patients’ *T*-score was lower at FN/TH than at LS [15]. However, clinical vertebral FFx are not always well ascertained. Radiographic vertebral fractural deformities are also difficult to be attributed to fragility causes. In fact, radiographic vertebral fractural deformities are not uncommon among subjects of normal BMD [64]. This is likely due to the fact that, compared with the long bones which have a thick cortex, vertebral cortex and endplates are much thinner and less resistant to external stress. To define the osteofrailia LS *T*-score threshold for older men will require other independent studies and more extensive analyses. Though there is no strong justification that the osteofrailia LS *T*-score would have to follow the osteofrailia FN *T*-score, before better evidence is available, a tentative “working” osteofrailia LS *T*-score threshold could also be ≤  − 2.0 for Caucasian men.

## Male–female differences in FFx TN /TH *T*-score tend to parallel the male–female B FH/TH *T*-score differences of the subjects without FFx

The MsOS (Hong Kong) baseline mean FN *T*-score among older Chinese women was − 1.76, and the MrOS (Hong Kong) baseline mean FN *T*-score among older Chinese men was − 1.25; thus, the difference was 0.51. Figure 3 shows that male hip FFx patients had a baseline FN *T*-score of 0.62 higher than those of female patients. In the statistical modeling shown in Fig. 4, the mean FN *T*-score among older US Caucasian community men was − 1.241, and the mean FN *T*-score among US older Caucasian community women was − 1.717; thus, the *T*-score difference was 0.476. Figure 2C shows that male–female differences in FFx TN/TH BMD tend to parallel the male–female BMD differences of the subjects without FFx. Figure 7BC shows the male female differences in LS BMD for clinical FFx patients and radiographic vertebral FFx patients tend to parallel the male–female LS BMD differences of the subjects without FFx. The mean *T*-scores being higher among community male population could be one of the important reasons that men fracture at higher *T*-scores and BMD than women, though Fig. 3 and Fig. 4 show the male–female FN *T*-score differences in community population are slightly less than male–female differences in FN *T*-score differences in hip FFx patients.

## Limitations of the current analyses

There are many limitations to the analyses in this article. The most important limitation is that the evidence to suggest the osteofrailia LS *T*-score threshold (− 2.0 for Caucasian men) remains limited. More studies are required to confirm or revise this osteofrailia *T*-score threshold for LS. While the evidence that older men suffer from hip FFx at higher FN/TH *T*-scores than those of older women is convincing, exactly by how much they differ requires more data which can only be made available with more future studies. The *T*-score data listed in Fig. 1 represent the results of a systematic literature search; the BMD data and the data of Wong et al. [34] only represent a random selection of literature. However, all the results are remarkably consistent. We did not study the biological or biomechanical reasons behind the FFx characteristics difference between men and women. As noted earlier, one of the important reasons may be that older men on average have higher *T*-scores than older women, with male–female FFx *T*-score differences largely parallelling the mean *T*-score differences between older community men and older community women. It is also possible that men with hip FFx could be involved on average higher level of trauma energy than women; however, the clinical endpoint is the same, i.e., hip FFx. Age is another important contributor for FFx risk. We checked the ages of the patients described in the reports we analyzed in the article, and we cannot attribute the difference in *T*-score mainly due to the age factor (Table 3). In the MrOS Hong Kong and MsOs Hong Kong follow-up studies, the baseline age was 0.2 years older for men than for women, the mean follow-up was 1.1 years longer for men than for women, and 69 and 63 hip FF were recorded among female participants and male participants respectively. The proportion of male patients relative to female patients was high for this particular study. However, the male–female difference in FN *T*-score of the Hong Kong studies was consistent with all other studies. We take the assumption that European older patients [17–19, 30–33, 35] were Caucasians. Though individual reports did not clarify how many percentages of their patients were Caucasians, we anticipate such an assumption is appropriately true, and the results from different studies were remarkably consistent. Finally, how this osteofrailia *T*-score can be integrated with other clinical parameters to predict FFx in real-world scenarios will require additional studies.

**Table 3:** The ages of males and female FFx patients for the studies listed in the current article. Ages are presented in mean and standard deviation or range. Ho et al. [22] only reported the mean ages

Data source	Age in years	
	Females	Males
Yeo et al. [18]	80 (74 ~ 87) (*n* = 91)	75 (68 ~ 83) (*n* = 21)
Vlachos et al. [19]	82.6 ± 7.1 (*n* = 51)	75.0 ± 11.3 (*n* = 19)
Lee et al. [20]	79.4 ± 9.4 (*n* = 819)	75.8 ± 10.4(*n* = 271)
Gani et al. [21]	78.3 ± 10.4 (*n* = 350)	74.0 ± 13.3 (*n* = 164)
Ho et al. [22]	82.2 (*n* = 167)	81.5 (*n* = 72)
Li et al. [23]	69.4 ± 9.0 (*n* = 268)	70.5 ± 12.9 (*n* = 92)
Wong et al. [34]	69.6 ± 8.4 (*n* = 47)	63.6 ± 7.3 (*n* = 16)
MrOS and MsOS Hong Kong	82.0 ± 5.9 (*n* = 69)	82.5 ± 5.7 (*n* = 63)
Wilson et al. [17]	69 (55 ~ 85) (*n* = 68)	68 (55 ~ 86) (*n* = 11)
Olszewski et al. [35]	79.7 ± 4.3 (*n* = 37)	72.6 ± 5.7 (*n* = 9)
Rathbun et al. [36]	81.0 ± 7.7 (*n* = 145)	80.4 ± 7.6 (*n* = 141)
Salimi et al. [37]	81.2 ± 7.7 (*n* = 128)	80.2 ± 7.3 (*n* = 117)
Mackey et al. [38]	74.1 ± 5.3 (*n* = 3211)	76.1 ± 6.5 (*n* = 346)
Domiciano et al. [39]	75.6 ± 5.9 (*n* = 29)	74.6 ± 6.0 (*n* = 7)

In summary, in this article we analyzed the observation that older men suffer from FFx at *T*-scores higher than older women. We propose a new category of lower BMD status for older men (i.e., “osteofrailia”), so that the same as the majority of female hip FFx occurs below the osteoporotic *T*-score, the majority of male hip FFx occurs below the osteofrailia *T*-score. The osteofrailia category for older community men is comparable in prevalence to the osteoporosis category for older community women. However, on average, older men below the osteofrailia *T*-score have only half the hip FFx risk as that of older women below the osteoporotic *T*-score. Detecting hip FFx high-risk male patients is important as multiple studies showed substantially higher post-FFx mortality among males than among females.
